# Kinetics of Alkali–Silica Reaction: Application to Sandstone

**DOI:** 10.3390/ma17122956

**Published:** 2024-06-17

**Authors:** Yongfu Yang, Min Deng, Liwu Mo, Wei Li

**Affiliations:** 1 College of Materials Science and Engineering, Nanjing Tech University, Nanjing 210009, China; hn_yyf@163.com (Y.Y.);; 2 School of Materials Science and Engineering, Luoyang Institute of Science and Technology, Luoyang 471023, China; 3 State Key Laboratory of Materials-Oriented Chemical Engineering, Nanjing 210009, China

**Keywords:** alkali–silica reaction, kinetics, sandstone, rock prism, mechanism of ASR expansion

## Abstract

Despite extensive research, the relationship between the progression of the alkali–silica reaction (ASR) and the expansion of concrete due to ASR, particularly for the heterogeneous aggregate with slow reactivity, is not thoroughly understood. In this paper, the dissolution kinetics of reactive silica present in sandstone when exposed to NaOH solutions, alongside the expansion characteristics of rock prisms under ASR conditions, were studied. The experimental results indicate that ASR behaves as a first-order reaction, accompanied by an exponential decrease in the concentration of OH^−^ over time, and the dissolution rate of silica is predominantly governed by diffusion dynamics. Notably, increasing the temperature accelerates ASR, which augments the expansive pressure in a confined and limited space, leading to more significant aggregate expansion. Conversely, higher temperatures also result in a diminished retention of ASR gels within the aggregate, leading to the mitigation of ASR expansion. Our findings underscore that larger aggregates retain a greater quantity of gels, resulting in more pronounced expansion. To establish an ASR prediction model based on the relationship of the ASR expansion of concrete to high and low temperatures, the parameters such as the range of curing temperatures and the grading size of aggregates should be carefully considered for the experiments.

## 1. Introduction

The alkali–silica reaction (ASR) is a complex chemical reaction taking place between the reactive silica in the aggregate and hydroxyl ions (OH^−^) from the pore solution in concrete [1,2]. ASR gel may adsorb water and cause the expansion and cracking of concrete. In general, two steps are involved in the ASR process: (1) the chemical reaction to form ASR gel, and (2) the expansion of gel due to the uptake of water [3]. Correspondingly, the ASR expansive behavior of concrete depends on: (1) chemical factors influencing the chemical reaction rate and degree, such as temperature [4,5], pore solution chemistry [6,7], and the reactivity of silica; (2) the response of physical factors to expansive pressure, such as the confined conditions of ASR gel [8,9,10,11], the distribution of reactive silica in the aggregate [12], and the rheological behavior of the gel [13]. Therefore, it is necessary and important to comprehensively understand the influence factors and expansion mechanism of ASR for establishing the ASR prediction model.

### 1.1. Chemistry of Alkali–Silica Reaction

The kinetics is equally important because the rate at which silica dissolves in the pore solution can largely control the overall rate of ASR. The explanation of the chemical process was given by Glasser et al. [14], and in more detail, by Bulteel et al. [15]. The topochemical rupture of the siloxane bonds possibly acts as follows:

(1) $$\equiv \;\mathrm{Si}\;\text{-}\;O\;\text{-}\;\mathrm{Si}\;\equiv +\;2OH^{-}\Leftrightarrow 2(\equiv \;\mathrm{Si}\;\text{-}\;O^{-})+H_{2}O$$

But it may occur in two steps [15]:

(1) The formation of Q3 sites due to siloxane bonds first breaking up by hydroxide ion attack, as follows:

(2) $${2\mathrm{SiO}}_{2}+{\mathrm{OH}}^{-}\to {\mathrm{SiO}}_{5/2}^{-}+{\mathrm{SiO}}_{5/2}H$$

(2) The dissolution of silica due to continued hydroxide ion attack on the Q3 sites to form silica ions, as follows:

(3) $${\mathrm{SiO}}_{5/2}^{-}+{\mathrm{OH}}^{-}+\frac{1}{2}HO_{2}\to H_{2}{\mathrm{SiO}}_{4}^{2-}$$

As the reactive silica structures (≡Si-OH, ≡Si-O-Si≡) gradually break down, they also attract the soluble alkali hydroxides (sodium hydroxide, NaOH, and/or potassium hydroxide, KOH), then form the hydrated alkali–silicate gel within the aggregate. Dron et al. [16] argued that ASR involves mainly ionic reactions in the pore solution. Different chemical equilibriums are possible, depending on the pH of the pore solution and the ionic species in the solution. Balachandran et al. [17] proposed a three-stage behavioral model to explain the dynamic relationship between ASR progression and pore solution composition. A high calcium ASR product (C–S–H- type gel) or a low calcium ASR product (ASR-type gel) was dictated by their accessibility to the pore solution.

The dissolution rate of silica is influenced by various factors, such as the pH, temperature, surface area of the reactive silica, the reactivity of silica, and the concentrations of K^+^, Na^+^, and Ca^2+^ present in concrete [18,19]. Dron et al. [20] studied the kinetics of the dissolution of opal in NaOH solution according to the conditions described in ASTM C289 [21]. Bulteel [15] and Verstraete et al. [22] quantified the reaction degree of flint dissolved in 0.79 mol/L KOH solution at different temperatures. Maraghechi [23] studied the dissolution rate of soda–lime–silica glass, and the results showed that it is highly related to both pH and temperature. Up to pH = 14 and at 60 °C, the dissolution rate of glass was found to be a function of [OH^−^]^0.2^. Kim et al. [19] investigated the chemical reaction sequence and kinetics of ASR by exposing α-cristobalite to 0.7 mol/L KOH or NaOH with the presence of solid calcium hydroxide (Ca(OH)_2_). They subsequently presented the results on the kinetics of the chemistry of the pore solution in a cementitious system containing the highly reactive aggregate [24].

### 1.2. Effect of Aggregate Properties

Whether or not concrete structures suffer ASR damage during service is fundamentally determined by the content of reactive silica in the aggregates and their properties. Aggregates are commonly classified as: non-reactive, moderately reactive, highly reactive, or very highly reactive, according to the results of standardized tests (e.g., the accelerated mortar bar test (AMBT, ASTM C1260 [25]) or the concrete prism test (CPT, ASTM C1293 [26])). Amorphous SiO_2_ (e.g., opal, natural, or synthetic glass) is known to be the most reactive, followed by metastable crystals (e.g., cristobalite and tridymite), microcrystalline silica, and other crystalline forms containing many lattice defects, residual strains, or internal microcracks. If the aggregate particle is composed entirely of reactive silica, such as opal or glasses, which have uniform composition throughout their volume, the aggregate surface may react quickly with the alkaline pore solution to form ASR gel at the surface. Onion skin cracks can be observed when alkali ions diffuse uniformly into the highly reactive aggregate [27,28]. Ichikawa et al. [27] proposed a reaction rim model to describe the ASR evolution. However, if the reactive silica is absent at the surface and is mostly contained as fine reactive particles distributed within a non-reactive matrix of the aggregate, such as many sandstones, greywackes and siliceous limestones, it takes time for the alkaline pore solution to penetrate into and contact with the reactive silica. As a result, the reaction proceeds more slowly, and the gel forms within the aggregate, leading to sharp cracks throughout the aggregate. As a type of slow-reacting aggregate, the mineralogy and fabric of rocks are responsible for different manifestations of the alkali–silica reaction [29,30]. Based on the results, Dunant et al. proposed a gel pocket model [31]. In the gel pocket model, expansive pressure forms at the random location of a reactive site (gel pocket), i.e., the origin of expansion is randomly distributed inside the aggregate [28,32]. 

The porosity degree of aggregate particles also plays a vital role in the rate of ASR expansion. According to the study by Ben et al. [30], it was reported that highly porous aggregates suffered more ASR expansion as compared to the aggregates with lower porosity. It was found that the alkali ions are more easily diffused in the porous aggregate particles than in the less porous aggregates, which then initiates aggregate dissolution leading to ASR. 

The kinetics of the highly reactive aggregates, such as opal, glass, flint, and α-cristobalite, has been studied by many researchers, while for the slow-reacting aggregates, the reactive silica randomly distributed within the non-reactive matrix of the aggregates, few studies have focused on the kinetics of silica dissolution. On the other hand, for the same aggregate, the expansion of ASR gel at different temperatures and aggregate sizes varies. The cause of the expansion induced by ASR gel remains controversial. In this paper, the kinetics of ASR in sandstone was investigated, and the corresponding induced expansion was discussed.

## 2. Materials and Methods

### 2.1. Materials

**Figure 1:**
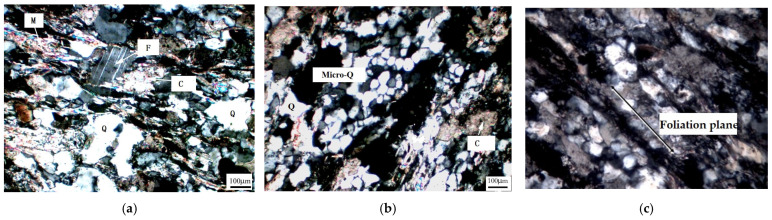
Petrographic analysis of sandstone with a crossed polarizer. (**a**) Clastic texture of sandstone; (**b**) microcrystalline quartz aggregated in sandstone; (**c**) direction of foliation planes. Q—quartz; C—calcite; F—feldspar; M—mica.

**Figure 2:**
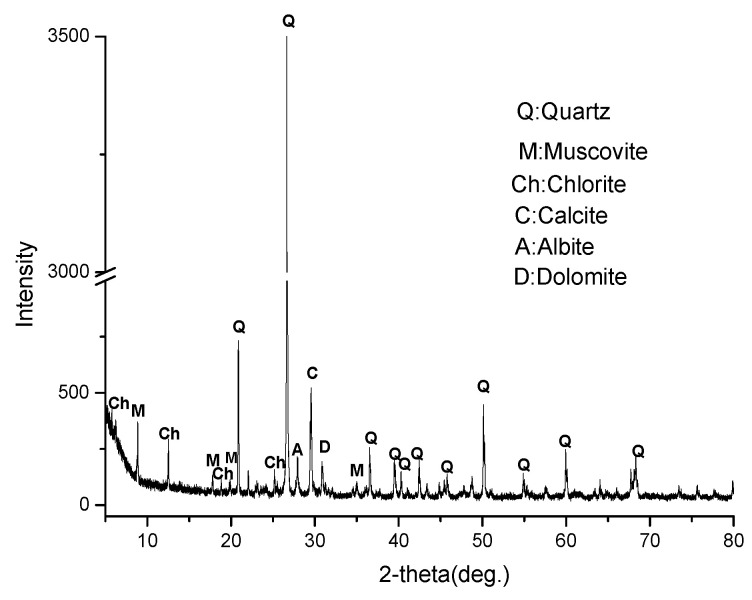
XRD pattern of the sandstone.

Sandstone, a clastic sedimentary rock, was used in this study, which also adopted the dam concrete for the Jinping Hydroelectric Project located at Sichuan Province, China, as the coarse aggregate. The sandstone is mainly composed of 72.0 wt.% SiO_2_, 11.2 wt.% Al_2_O_3_, 4.5 wt.% Fe_2_O_3_, 1.4 wt.% CaO, 3.6 wt.% MgO, 2.0 wt.% K_2_O, and 1.0 wt.% Na_2_O; and the ignition loss is 4.1 wt.%. The rock exhibits a mosaic structure consisting of quartz, mica, feldspar, calcite, and chlorite, according to the petrographic analysis using a LEICA DM750P Polarization Microscope (Leica Microsystems, Tokyo, Japan) (Figure 1a). Microcrystalline quartz is considered to be the reactive phase, which distributes as an aggregate in the rock (Figure 1b). The amount of microcrystalline quartz in the rock is approximately 10 wt.%. A continuous foliation can be distinguished, and the direction of the foliation planes is shown by a black arrow in Figure 1c. Minerals in the rock were also confirmed by X-ray diffraction (XRD, Bruker D8 Focus, Billerica, MA, USA, Cu, 40 kV, 40 mA, 5°/min, ), and the results are shown in Figure 2. The expansion of the mortar was 0.19% at 14 days according to the accelerated mortar bar method (ASTM C1260 [25]), and the expansion of the concrete prisms was 0.04% at 1 year according to ASTM C1293 [26]. This indicates that sandstone is potentially alkali-reactive, and that it is a slow-reacting aggregate. Chemical grade NaOH (NaOH ≥ 95%) was used to prepare alkali solutions with deionized water.

### 2.2. Experimental Methods

#### 2.2.1. Kinetics of Silica Dissolution 

The extent of the reaction between the NaOH solution and sandstone was determined using ASTM C289, while some conditions were adjusted: (1) The crushed sandstone particles with the size fraction of 0.315–0.630 mm instead of that of 0.15–0.3 mm, described in ASTM C289, were used in the experiment. (2) The reaction conditions, such as different temperatures (40 °C, 60 °C, and 80 °C) and the different concentration of NaOH solution (1.0 mol/L, 0.5 mol/L, 0.3 mol/L and 0.2 mol/L), were used in the experiment. (3) The duration of the reaction was prolonged to 90 days.

Two batches of test specimens were prepared for the experiment. One is used for investigating the influence of temperatures designated at 40 °C, 60 °C, and 80 °C, respectively, and the concentration of NaOH solution is 1.0 mol/L. Another batch of specimens is used for investigating the influences of NaOH concentration, for which the concentrations are set to 0.5 mol/L, 0.3 mol/L, and 0.2 mol/L, respectively, at a specific temperature of 60 °C. A total of 25.0 g of sandstone particles was placed into a polypropylene copolymer container. Prior to the mixing of sandstone and the NaOH solution, they were separately preheated to the reaction temperature. Following this, 25 mL of NaOH solution was added into the container. The containers were sealed and stored at the preset temperature conditions.

ASR products are present in two forms: (1) free silica ions in alkali solution, and (2) precipitated silica to form sodium silica hydrates (N-S-H, ASR gels). At the end of the exposure periods, the container was cooled immediately, and the liquid was filtered. The filtrates were collected, and the content of dissolved silica and the reduction in alkalinity were detected according to the method described in ASTM C289. The residues were dried at 105 °C for 24 h, then ground into powders by passing them through a 0.045 mm sieve. A total of 5.0 g of the powders were immersed in 500 mL of 1:1 dilute hydrochloric acid (HCl) and stirred for 0.5 h [19]. This acid treatment was intended to dissolve the ASR gels in the solids and dilute the silicate ions for silica content detection. The dissolution solution was filtered, and the content of dissolved silica was measured, according to ASTM C289. 

The reaction degree of the silica was calculated at different ages. The reaction degree α is defined as:

(4) $$\alpha =\frac{{Si}_{dissolved}}{{Si}^{\infty }}=\frac{{Si}_{solution}+{Si}_{gel}}{{Si}^{\infty }}$$

where ${Si}_{solution}$ is the amount of free silicate ions in alkali solution at time *t*, ${Si}_{gel}$ is the amount of ASR gels, ${Si}^{\infty }$ is the asymptotic value for *t* = ∞, of which the value was obtained through curve-fitting, described by Gao [33]. The ${Si}^{\infty }$ is 2.4 g in the reaction system with 25.0 g of sandstone.

#### 2.2.2. Expansion of Rock Prism

The test method for potential alkali reactivity of carbonate rocks was used for reference [34], but some parameters were altered in this experiment: (1) Sandstone instead of carbonate rock was used in the rock prism test method [35]; (2) Rock prisms of 10 mm × 10 mm × 35 mm and 20 mm × 20 mm × 60 mm instead of cylinder sample Φ9 mm × 35 mm were prepared. Each batch of rock prisms contains three mutually perpendicular specimens. Stainless steel reference studs were fixed on the mid-points at the end faces of the rock prisms. Specimens were immersed in deionized water at a room temperature (22 ± 1 °C), and the length was measured at different intervals until the change in length in a 24 h water immersion period did not exceed 0.02%. The stable datum is used as the initial length of rock prism. Following this, the specimens were immersed in 1 mol/L NaOH solution and cured at 60 °C and 80 °C, respectively. The ratio of the NaOH solution to the solid (by mass) is 3. When the concentration of OH^−^ ions of the alkali solution was less than 0.7 mol/L, an NaOH tablet was added to improve the concentration of OH^−^ ions to 1.0 mol/L [35]. Prior to the length measurement, the specimens were removed and cooled down to the room temperature (22 ± 1 °C). The length change was calculated as follows:

(5) $$∆l=\frac{l_{i}-l_{0}}{l_{0}}\times 100\%$$

where ∆l is the length change at the test age, *l*_i_ is the length in mm at the test age, and *l*_0_ is the initial length after equilibrium in water. In the three mutually perpendicular specimens, the max expansion specimen was used for plotting.

## 3. Results and Discussion

### 3.1. Kinetics of ASR in Sandstone

#### 3.1.1. Reaction Degree of Silica in Sandstone

As mentioned previously, the total dissolved silica consisted of (1) free silicate ions in solution, and (2) precipitated silica as ASR gels. A specific reaction degree α_1_ is defined as follows:

(6) $$\alpha_{1}=\frac{{Si}_{solution}}{{Si}^{\infty }}$$

α_2_ is determined by the difference between total dissolved silica and free silica in solution α_1_:

α_2_ = α − α_1_ (7)

**Figure 3:**
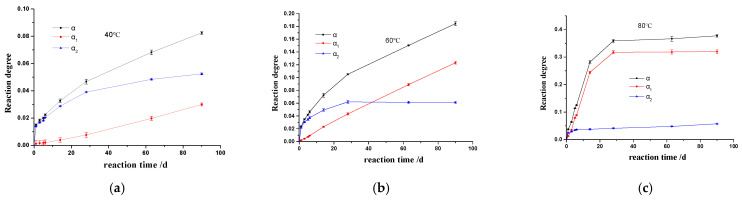
Reaction degree α as a function of time at different temperatures. (**a**) 40 °C, (**b**) 60 °C, (**c**) 80 °C.

The reaction degree of sandstone immersed in 25 mL 1 mol/L NaOH solution, cured at 40 °C, 60 °C, and 80 °C, respectively, is given in Figure 3.

At 40 °C, the reaction degree α was increased continuously in the experimental period. In the early stages, α increased rapidly, which was mainly due to the rapid increase in α_2_. After a short plateau at 6 days, α_1_ increased steadily. When sandstone was immersed in 1 mol/L NaOH solution for 90 d, the α, α_1_, and α_2_ were 0.08, 0.03, and 0.05, respectively. At 60 °C, α increased continuously, which is similar to the reaction at 40 °C. The difference is that α_2_ was increased to reach an asymptotic value of 0.06. As the temperature increased to 80 °C, the silica dissolved in solution increased rapidly in 28 d, with the highest reaction degree of 0.38, and thereafter, it reached a plateau. The precipitated silica was increased rapidly in the early stages, and then reached a steady value of around 0.04. During the reaction period from 28 d to 90 d, the precipitated silica increased slowly, with the value of α_2_ increased from 0.04 to 0.06.

#### 3.1.2. Dissolution Rate Constant

The dissolution of silica is influenced by many factors, such as mineral type of silica, particle size, surface area, pH value, and composition of the solution [36]. In this model, it is assumed that the formation and dissolution of ASR gel is a dynamic equilibrium process, which simplifies the diffusion process of ions in the alkali silica reaction system. The rate of a solid–liquid reaction is usually controlled by chemical dynamic or diffusional dynamic. The chemical control model is usually described as follows [37]:

(8) 1−1−α13=krt

And the diffusional control model is described as [37]:

(9) 1+21−α−31−α23=kdt

where *α* is the reaction degree, *k* is the dissolution rate constant, and *t* is the reaction time. To determine the experimental dissolution rate constants (*k*), the relationships between the reaction degree (*α*) and the time (*t*) were explored according to Equations (8) and (9) for the reaction systems at 40 °C, 60 °C, and 80 °C, respectively. The results are listed in Table 1.

As shown in Table 1, the values of kd=1+21−α−31−α23t at a certain temperature are roughly equal, while the values of kr=1−1−α13t are discrete. The standard deviation values (SD) via ANOVA are listed in Table 2.

**Figure 4:**
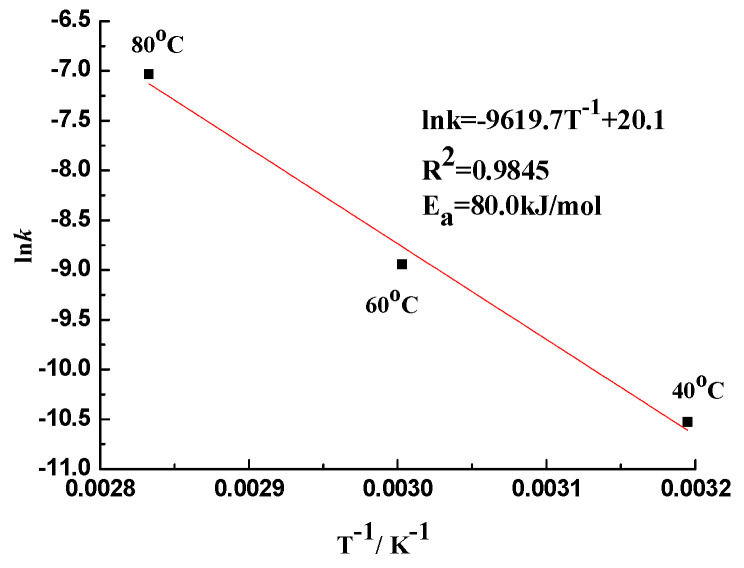
Arrhenius plot for the reacting systems with sandstone immersed in 1 mol/L NaOH.

Obviously, the SD of *k_r_* is much greater than that of *k_d_* at each temperature. This means that the rate of silica dissolved in alkali solution is controlled by the diffusional dynamic. The average value of $k_{d}$ (only the roughly equal values) represents the dissolution rate constants (*k*) for the reaction system at each temperature. The influence of temperature on the dissolution rate of the silica in sandstone was examined using the Arrhenius equation, and the results are shown in Figure 4. The activation energy (*Ea*) for the dissolution of silica in sandstone is 80.0 kJ/mol. The value is a little higher than that for flint (78 kJ/mol) [15] and α-cristobalite (73.05 kJ/mol) [19].

#### 3.1.3. Reaction Order

To determine the order of the alkali–silica reaction, only the [OH^−^] was changed for all of the reaction systems. Assuming the reaction order is *n*, the kinetics of the reaction could be described as:

(10) $$r\propto k{\left[{OH}^{-}\right]}^{n}$$

where *r* is the rate of reaction, *k* is the reaction rate constant depending on the reaction temperature, and n is the reaction order. The following relationship can be deduced from Equation (10) when the temperature is fixed:

(11) $$lgr\propto nlg[{OH}^{-}]$$

**Figure 5:**
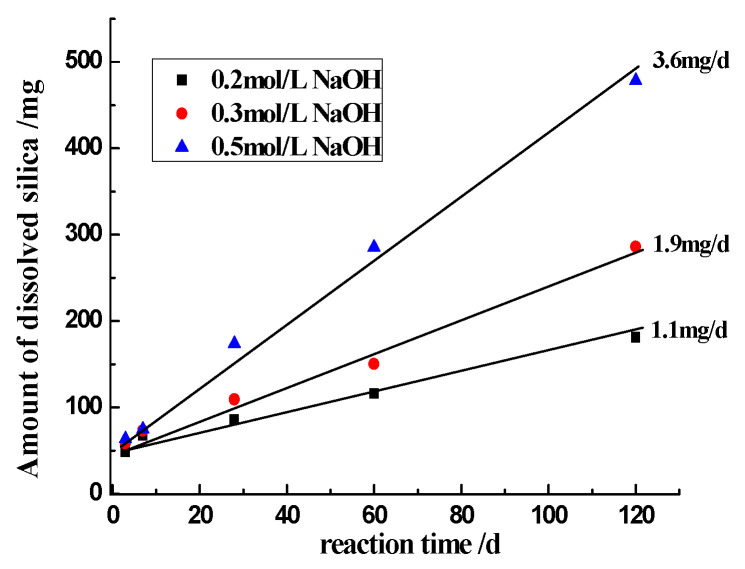
The reaction rate of silica in NaOH solution.

**Figure 6:**
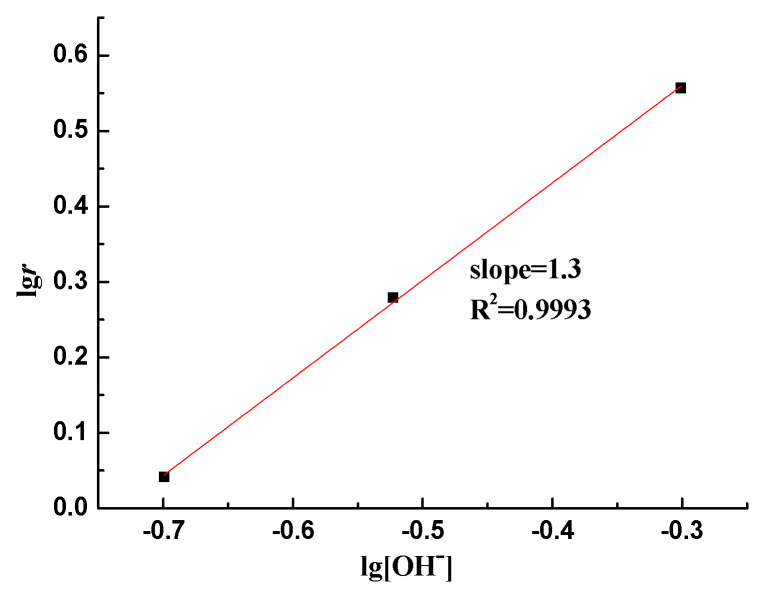
The order of alkali–silica reaction.

The influence of the concentration of NaOH on silica dissolution at 60 °C is shown in Figure 5. The amount of dissolved silica is the sum of silica in solution and the precipitated silica. The dissolved silica increases rapidly in the first 3 days, and it is mainly precipitated in inner part of sandstone particles. After that, at each concentration of NaOH, the amounts of dissolved silica are approximately proportional to the reaction time. Figure 6 shows the relationship between the magnitude of dissolved silica and the reaction time when different NaOH concentrations were used, and the slope of the fitted line to the different concentration of NaOH represents the reaction rate. The relationship between *r* and [OH^−^] was plotted according to Equation (11), shown in Figure 6. The slope represents the reaction order, *n*, which is 1.3, indicating that ASR is a first-order reaction. Obviously, the reaction rate is proportional to the [OH^−^].

### 3.2. Expansion of Rock Prism

**Figure 7:**
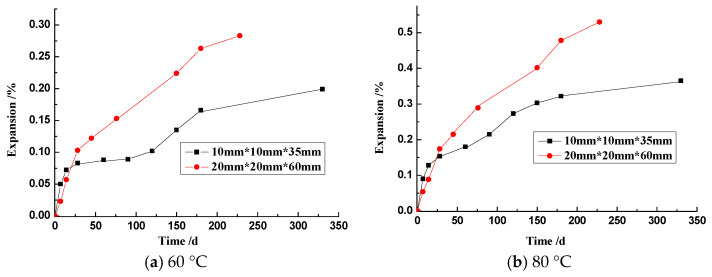
ASR expansions of rock prism cured in 1 mol/L NaOH solution at 60 °C and 80 °C.

The expansion of rock prisms with the size of 10 mm × 10 mm × 35 mm and 20 mm × 20 mm × 60 mm, cured in 1 mol/L NaOH at 60 °C and 80 °C, respectively, is shown in Figure 7. In the early ages, prisms of 10 mm × 10 mm × 35 mm showed a higher expansion at either 60 °C or 80 °C, while at the later stage, prisms of 20 mm × 20 mm × 60 mm showed a higher expansion. For the prisms of 10 mm × 10 mm × 35 mm, the expansions obtained in 330 days at 60 °C and 80 °C are 0.20% and 0.36%, respectively. For the prisms of 20 mm × 20 mm × 60 mm, the expansions obtained in 228 days at 60 °C and 80 °C are 0.28% and 0.53%, respectively. With increasing temperature, the expansion of the rock prism increased.

### 3.3. Discussion

The deterioration of concrete structures due to ASR expansion is usually initiated by the chemical reaction accompanying the formation of swelling gels. In sandstone, the microcrystalline quartz particles (reactive SiO_2_) are surrounded by other minerals (non-alkali-reactive matrix). The alkali solution diffuses into the sandstone through pores and other channels, and then the chemical reaction takes place at the surface of the microcrystalline quartz to form ASR gels. As the reaction progresses, the amount of ASR gel around the reactive silica sites increases. On the one hand, the ASR gels will diffuse and fill the diffusion channels, leading to a decrease in the diffusion coefficient. On the other hand, cracks generated by ASR expansion will enhance the diffusion coefficient. With the evolution of the reaction, gels increased within the sandstone, and they would create pressure if the gels formed in a confined and limited space, but the gels would exude from the confined space into the alkali solution to release the pressure, accompanied by an increase in free silica ions in the alkali solution. In addition, the reactant NaOH is continuously consumed, and the concentration of OH^−^ constantly decreases. Therefore, the diffusion of OH^−^ is not steady. In this model, it is assumed that the formation and dissolution of ASR gel is a dynamic equilibrium process, which simplifies the diffusion process of ions in the alkali silica reaction system.

#### 3.3.1. Effect of the Temperature

**Figure 8:**
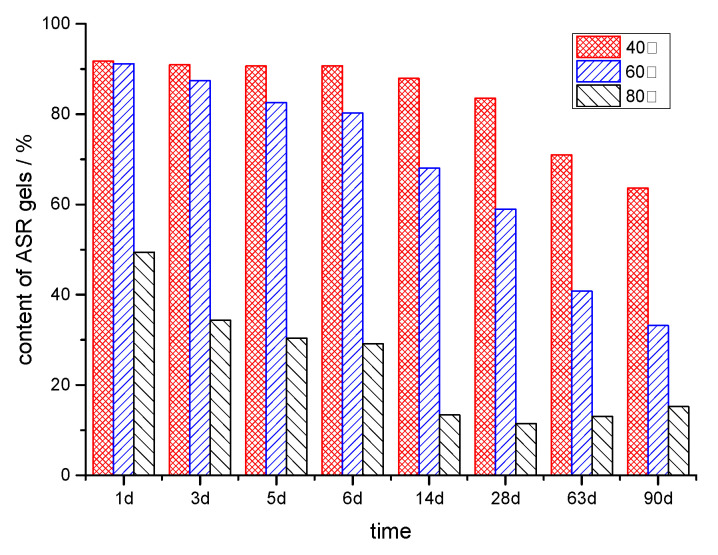
Content of ASR gels inside the particle at different temperatures.

The temperature plays an important role in the alkali–silica chemical reaction and ASR gel behavior in the sandstone aggregate. As a chemical reaction, the kinetics of ASR in sandstone follow Arrhenius’ law, which means that increasing the temperature may accelerate the process of ASR, forming more products. In many laboratory studies on assessing the performance of concrete due to ASR, it is common to use temperature elevation to accelerate the rate of reaction and to explore the correlation between the expansion of ASR at high and low temperatures for predicting the performance of concrete in service. It is worth noting that the ASR expansion is related to the reacted fraction of aggregates, but it is also affected by the amount of ASR gel retained in the aggregate and confined in a restricted space. As shown in Figure 3, in the early stage, α_2_ is greater than α_1_, which means that the amount of gel is always higher than that of the free silicate ions. As the reaction proceeds, the amount of silicate ions in the alkali solution increases gradually. Elevated temperature boosts the exudation of ASR gels from the sandstone into the alkali solution. Figure 8 shows the content of ASR gels inside the particle at different temperature, in which the content of gels is defined as α_2_/α.

With increasing temperature, the content of ASR gels decreased dramatically. At 40 °C, the reacted silica is mainly in the form of ASR gels, with an amount of 5% at 90 d, and the amount of gel were increased continuously. At 60 °C or 80 °C, the amounts of ASR gel retained in the sandstone came to a plateau, at 6% and 4%, respectively. Although more of the reactive silica reacted at a higher temperature, the products mostly exist in the form of silicate ions in the alkali solution. Research results show that high temperature decreases the viscosity of ASR gel [13,38], which means that the higher temperature enhances the movement of ASR gel, making it more likely to flow out through the pores or cracks. The amount of ASR gels retained inside the aggregate decreased with the increase in temperature, which will reduce the ASR expansion [39].

#### 3.3.2. Effect of Hydroxyl Ions

**Figure 9:**
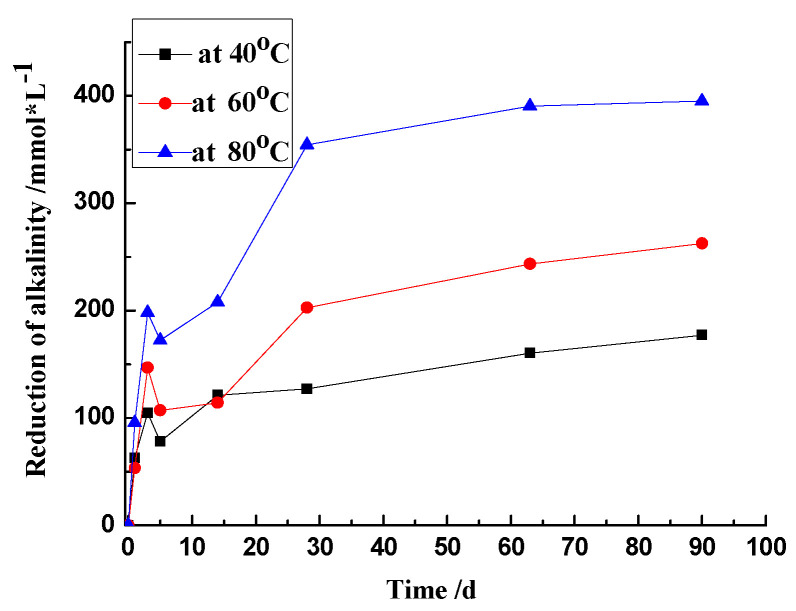
Reduction of alkalinity of 1 mol/L NaOH reacted with sandstone.

According to the chemical reaction described in Equations (2) and (3), the hydroxyl ions play a vital role in the dissolution rate of silica, since it acts as a reactant attacking the reactive silica. The results show that the dissolution of reactive silica within sandstone attacked by OH^−^ ions can be expressed as a first-order reaction, which means that the dissolution rate of reactive silica is only proportional to the concentration of OH^−^ to the power of one. As ASR proceeds, the hydroxide ions consume continuously, and the [OH^−^] reduces gradually. The drop in alkalinity is shown in Figure 9.

During the first 3 days, the concentration of OH^−^ ions dropped rapidly at every temperature, which might be attributed to the rapid alkali–silica reaction. At the later stages, the decline in the concentration of OH^−^ ions became slow. It should be noted that the [OH^−^] in the solution at 80 °C remained around 0.6 mol/L, even though the reaction degree of silica reached a plateau. It can thus be inferred that the reaction in the system with 25.0 g of sandstone and 25 mL of 1 mol/L NaOH did not come to end, but to an equilibrium of dissolution.

Therefore, the effect of OH^−^ ions could be described as follows: at the early stage, with a high concentration, OH^−^ ions rapidly attack the reactive silica, producing abundant gels, leading to a rapid ASR expansion; at the late stage, with a declining concentration, the reaction rate slows down, with a slow growth of ASR gels.

Ca(OH)_2_ presented in the alkali–silica reaction plays an important role in ASR expansion [40]. Struble et al. [41,42] reported that for a homogenous amorphous silica aggregate, the silica minerals merely dissolved and remained in the solution in the absence of calcium (Ca), while with the presence of Ca, the reaction rim of the aggregates was formed in a confined space and therefore, produced the expansion of concrete. Unlike the homogenous silica aggregate, sandstone itself provides restricted spaces for accommodating the expansive ASR gels. In the absence of Ca in this experiment, the rock prisms expand continuously (Figure 7), but it can be inferred that the presence of Ca(OH)_2_ accelerates the rate of ASR expansion. High-alkali and low-calcium silica hydrates [C-(Na/K)-S-H) with higher viscosity form on the surface of the aggregate, which can reduce the exudation of gels from the particles [10,13].

#### 3.3.3. Effect of Particle Size

**Figure 10:**
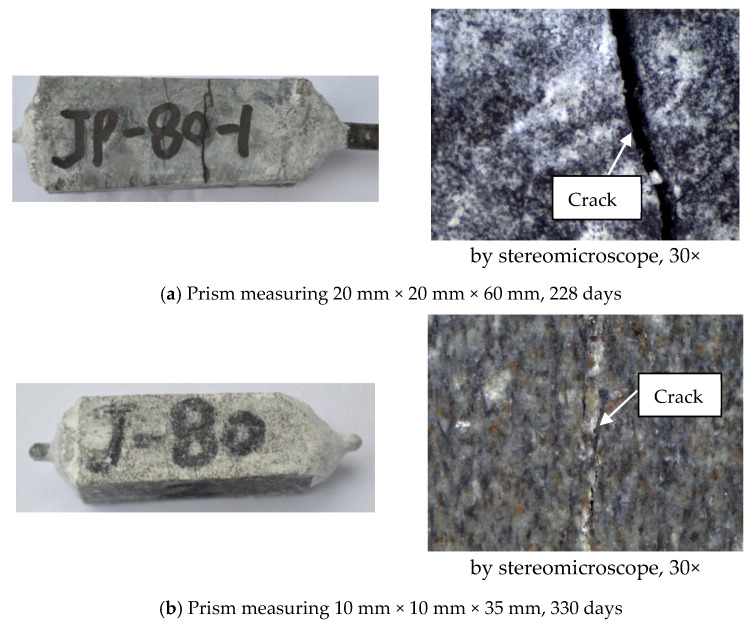
Cracks in the rock prisms cured in 1 mol/L NaOH at 80 °C.

**Figure 11:**
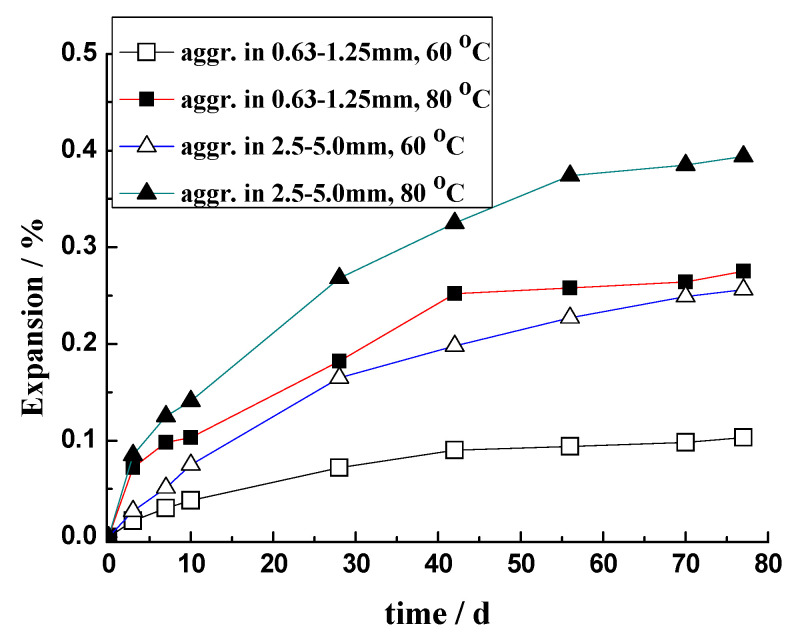
Expansion of mortar bar cured in 1 mol/L NaOH solution [43].

The amount of gel retained inside the aggregate is also influenced by the prism size. Figure 7 shows the ASR expansion of the sandstone rock prisms with different cross-sections. In the experimental system of the rock prism and 1 mol/L NaOH solution, with the increasing size of the rock prism, the expansion of the rock prism increased. Figure 10 shows the micrographs of rock prisms cured at 80 °C. Obviously, ASR damage to the 20 mm × 20 mm × 60 mm prism seems more serious. Ke [43] performed an experiment using the same sandstone as that used in this study. In Ke’s experiment [43], the aggregates with sizes of 0.63–1.25 mm and 2.5–5.0 mm were used, respectively, and mortar bars with 20 mm × 20 mm × 80 mm were cast. The effect of the curing temperature on the ASR expansion of mortar bar was explored, and the results are shown in Figure 11. At the same temperature, the expansion of mortar bar with 2.5–5.0 mm aggregates is usually greater than that of the mortar bar with the 0.63–1.25 mm aggregates. Larger aggregates tends to retain a higher amount of gels inside for the longer distance of diffusion, leading to a higher expansion and more serious damage.

## 4. Conclusions

The study of the kinetics of the alkali–silica reaction (ASR) in sandstone has provided significant insights into the dissolution behavior of reactive silica under varying conditions, and the following conclusions can be drawn:

(1) The ASR of sandstone is a first-order reaction. The alkali–silica reaction in sandstone conforms to a first-order kinetic model. The reaction rate is directly proportional to the concentration of hydroxide ions ([OH^−^]) and decreases exponentially over time as the OH^−^ ions are consumed. This indicates that without the introduction of new hydroxide ions, the silica dissolution rate will progressively diminish. The reaction kinetics adhere to the principles outlined by Arrhenius’ law, demonstrating that temperature is a critical factor in the reaction rate.
(2) There is a temperature influence on ASR dynamics. Elevated temperatures significantly accelerate the ASR process, increasing the rate of silica dissolution and subsequent gel formation. However, higher temperatures also reduce the retention of ASR gels within the aggregate particles, which mitigates the overall expansion caused by the reaction. This dual effect underscores the complexity of predicting ASR behavior solely based on temperature. Larger aggregates tend to retain more ASR gels, leading to greater expansion, which is a crucial consideration for understanding and modeling ASR in real-world scenarios.
(3) The implications for predictive modeling are as follows: The study highlights the necessity of incorporating various parameters, such as temperature and aggregate size, into predictive models for ASR. These factors must be meticulously controlled in experimental setups to ensure the accuracy and applicability of the models. The relationship between ASR expansion at different temperatures and the structural properties of the aggregate provides a basis for developing more reliable predictive tools for concrete deterioration due to ASR.

This study primarily focuses on the behavior of sandstone aggregates under controlled laboratory conditions. While the insights are valuable, real-world conditions often present additional variables, such as fluctuating environmental temperatures and varying chemical compositions of concrete. Future studies should aim to replicate these conditions to further validate the findings. By addressing these aspects, the understanding and management of ASR in concrete can be significantly improved, leading to a more durable and resilient infrastructure.

## Figures and Tables

**Table 1:** Values of dissolution rate constant (k) at 40 °C, 60 °C, and 80 °C, respectively.

Time/Days	40 °C			60 °C			80 °C		
	α	$k_{r}$	$k_{d}$	α	$k_{r}$	$k_{d}$	α	$k_{r}$	$k_{d}$
1	0.015	5.071 × 10^−3^	7.842 × 10^−5^	0.024	7.932 × 10^−3^	1.902 × 10^−4^	0.050	1.690 × 10^−2^	8.526 × 10^−4^
3	0.018	2.054 × 10^−3^	3.844 × 10^−5^	0.035	3.907 × 10^−3^	1.375 × 10^−4^	0.085	9.724 × 10^−3^	8.375 × 10^−4^
5	0.020	1.347 × 10^−3^	2.749 × 10^−5^	0.042	2.827 × 10^−3^	1.196 × 10^−4^	0.114	7.887 × 10^−3^	9.110 × 10^−4^
6	0.022	1.257 × 10^−3^	2.869 × 10^−5^	0.046	2.620 × 10^−3^	1.230 × 10^−4^	0.125	7.268 × 10^−3^	9.253 × 10^−4^
14	0.033	7.882 × 10^−4^	2.614 × 10^−5^	0.072	1.768 × 10^−3^	1.296 × 10^−4^	0.282	7.462 × 10^−3^	2.178 × 10^−3^
28	0.047	5.657 × 10^−4^	2.676 × 10^−5^	0.105	1.298 × 10^−3^	1.384 × 10^−4^	0.359	4.918 × 10^−3^	1.847 × 10^−3^
63	0.068	3.693 × 10^−4^	2.549 × 10^−5^	0.150	8.384 × 10^−4^	1.284 × 10^−4^	0.366	2.240 × 10^−3^	8.598 × 10^−4^
90	0.082	3.141 × 10^−4^	2.623 × 10^−5^	0.184	7.291 × 10^−4^	1.375 × 10^−4^	0.377	1.623 × 10^−3^	6.421 × 10^−4^

**Table 2:** SD of dissolution rate constant (*k*) at 40 °C, 60 °C, and 80 °C, respectively.

	SD		
	40 °C	60 °C	80 °C
*k_r_*	0.00157	0.00236	0.00481
*k_d_*	1.8 × 10^−5^	2.2 × 10^−5^	5.5 × 10^−4^

## Data Availability

Data are contained within the article.
